# Mitigation of Cerebral Ischemia-Reperfusion Injury in Rat by Phellopterin via Antioxidant and Anti-inflammatory Mechanisms

**DOI:** 10.5812/ijpr-163643

**Published:** 2025-09-16

**Authors:** Huici Wang, Huan Wang

**Affiliations:** 1 Department of Neurology, Xingtai People's Hospital, Xingtai, China; 2 Department of Clinical Laboratory, Xingtai People's Hospital, Xingtai, China

**Keywords:** Cerebral Ischemia-Reperfusion Injury, Phellopterin, Antioxidant, Anti-inflammatory, Mechanism

## Abstract

**Background:**

Cerebral ischemia-reperfusion (I/R) injury is a significant pathological process in ischemic stroke, marked by oxidative stress, inflammation, and neuronal damage once blood flow is restored.

**Objectives:**

The present study investigates the neuroprotective effects of phellopterin, a natural coumarin derivative, in a rat model of cerebral I/R injury.

**Methods:**

The I/R injury was induced using the middle cerebral artery occlusion (MCAO) method, subjecting rats to 120 minutes of ischemia followed by 24 hours of reperfusion. Phellopterin was administered intragastrically at doses of 0.5 and 2.0 mg/kg, based on prior studies indicating effective therapeutic ranges in preclinical models. Neurological deficit scores (NDS) assessed functional impairments. Molecular markers of oxidative stress, including malondialdehyde (MDA) and superoxide dismutase (SOD), alongside inflammatory cytokines such as tumor necrosis factor-alpha (TNF-α) and interleukin-6 (IL-6), were measured using enzyme-linked immunosorbent assays (ELISA). Gene expression analysis utilized quantitative reverse transcription polymerase chain reaction (RT-PCR) to evaluate mRNA levels of key oxidative stress and inflammatory markers.

**Results:**

Our results indicated that phellopterin significantly reduced NDS in a dose-dependent manner. Molecular analyses demonstrated decreased MDA levels and increased SOD activity, reflecting reduced oxidative damage, alongside lowered TNF-α and IL-6 levels, indicating suppressed inflammation. The RT-PCR confirmed the upregulation of antioxidant genes [nuclear factor erythroid 2-related factor 2 (Nrf2), heme oxygenase-1 (HO-1)] and downregulation of pro-inflammatory genes [nuclear factor kappa B (NF-κB), iNOS].

**Conclusions:**

In conclusion, phellopterin exhibits dual antioxidant and anti-inflammatory effects, suggesting its potential as a therapeutic agent for ischemic stroke. These findings underscore the potential of phellopterin as a promising candidate for the development of new treatments for ischemic stroke.

## 1. Background

Stroke is a critical global health issue, ranking among the top five causes of death worldwide. It occurs when the blood supply to the brain is interrupted in several ways. This disruption prevents neurons from receiving critical nutrients such as glucose, oxygen, and energy, eventually leading to neuronal death (1, 2). Remarkably, the restoration of blood flow and oxygen (reperfusion) can paradoxically exacerbate cellular injury in ischemic areas, referred to as cerebral ischemia/reperfusion (I/R) injury in the medical literature (3). The restoration of blood flow after a period of ischemia triggers a complex cascade of events, accompanied by increased detrimental oxidative reactions, activated inflammatory responses, and nerve cell injury. The interplay of these mechanisms leads to the cumulative damage observed in the affected brain (4).

Despite advancements in medical research, treatment strategies for the prevention and effective management of cerebral I/R injury remain limited. This underscores the urgent need for new interventions that target the fundamental mechanisms of this devastating disease (5). Given the central role of oxidative stress and inflammation in stroke pathophysiology, compounds with dual activity, such as phellopterin, are of particular interest. Several natural compounds, such as resveratrol, curcumin, and pterostilbene, have demonstrated neuroprotective effects against cerebral I/R injury through antioxidant and anti-inflammatory mechanisms (6, 7). These findings support further investigation into similar bioactive agents like phellopterin.

Phellopterin is derived from the well-known functional food and herb *Angelica dahurica* (8). It has demonstrated potential anti-inflammatory and antioxidant properties, two pivotal contributors to the pathophysiology of cerebral I/R injury (9, 10). Phellopterin is an ingredient used in traditional medicine, known for its anti-inflammatory and antispasmodic properties, and has previously demonstrated possible neuroprotective effects in several preclinical studies (11).

Phellopterin has shown therapeutic properties in multiple previous diseases. Xu et al. studied the effects and mechanisms of phellopterin on colitis-associated cancer (CAC). They identified decreased levels of pro-inflammatory markers, interleukin (IL)-6, IL-1β, and tumor necrosis factor (TNF)-α, and elevated levels of the anti-inflammatory marker IL-10. They also observed that colon tissue had decreased protein levels of toll-like receptor 4 (TLR4) and nuclear factor kappa B (NF-κB) p65 (12). In another study by Chen et al., they found that phellopterin was a bioactive compound with activities against type 2 inflammation and a signal transducer and activator of transcription 3 (STAT3) inhibitor, making it a promising active dermatological agent against atopic dermatitis (AD) (13). Takomthong et al. also demonstrated that phellopterin could ameliorate neuronal cell damage from H_2_O_2_ and amyloid beta (Aβ)1–42 toxicity in Alzheimer’s disease (14). Furthermore, Zou et al. demonstrated that phellopterin has anti-inflammatory effects both in vitro and in vivo, with a potential mechanism involving sirtuin 1 (SIRT1) in diabetic ulcers (10).

## 2. Objectives

Despite multiple studies demonstrating phellopterin’s anti-inflammatory and antioxidant properties in various disease contexts, such as CAC, AD, Alzheimer’s disease, and diabetic ulcers, no prior investigation has evaluated its effects in cerebral I/R injury. This gap in the literature underscores the novelty of the present study, which, to our knowledge, is the first to demonstrate phellopterin’s neuroprotective potential in this condition. Here, we investigated the neuroprotective effect of phellopterin, a natural coumarin derivative, on cerebral I/R injury in a rat model.

## 3. Methods

### 3.1. Animals

A priori power analysis (α = 0.05, power = 0.8) was conducted using G*Power software, estimating that a minimum of 8 animals per group would detect a large effect size (Cohen’s d = 1.2) in outcome measures such as neurological deficit scores (NDS). We included 10 rats per group to account for potential attrition and ensure sufficient statistical power. A total of thirty healthy male Sprague-Dawley (SD) rats, aged 6 to 8 weeks and weighing approximately 200 ± 20 g, were kept under controlled conditions with a meticulously regulated environment with a stable temperature of 24°C and a light/dark cycle of 12 hours. They had unrestricted access to both food and water. Animals were housed in groups of three to four per cage with wood-chip bedding, nesting material, and cardboard tunnels for enrichment. Food and water were available ad libitum. Health status, grooming, mobility, and feeding behavior were monitored daily by trained staff, in accordance with ARRIVE 2.0 guidelines.

### 3.2. Cerebral Ischemia-Reperfusion Injury Model and Treatment Protocol

As previously outlined (15), cerebral I/R injury was induced in rats through middle cerebral artery occlusion (MCAO). Anesthesia was administered using ketamine (80 mg/kg) and xylazine (10 mg/kg). Once the rats were fully unconscious, a midline cervical incision was made to expose the right common carotid artery. An A4-0 nylon filament with a circular tip was then inserted from the common carotid artery to the internal carotid artery to occlude the right middle cerebral artery. After two hours of occlusion, the nylon filament was carefully removed to allow for reperfusion of the MCA for 24 hours. Animals were randomly assigned to groups using a computer-generated randomization sequence. Investigators conducting behavioral scoring, tissue processing, and data analysis were blinded to group allocations throughout the study. The individual assessing NDS was blinded to treatment allocation; tissue samples were labeled with anonymized codes before molecular assays; and molecular analyses [enzyme-linked immunosorbent assays (ELISA) and reverse transcription polymerase chain reaction (RT-PCR)] were performed by a separate investigator who remained blinded to group identities until all data were collected and finalized. The rats were randomly assigned to three groups: Cerebral I/R control + sterile phosphate-buffered saline (PBS), cerebral I/R + 0.5 mg/kg phellopterin (Biosynth, USA), cerebral I/R + 2 mg/kg phellopterin (12) by intragastric administration. The vehicle control group received an equivalent volume (1 mL/kg) of sterile PBS intragastrically, administered at the same time points as the phellopterin treatment groups.

### 3.3. Neurological Deficit Scores

The NDS in rats were assessed 24 hours after MCAO using the Bederson Neurological Scale (16), which is categorized as follows: No neurological deficits (0); inability to fully extend the left forepaw (1); reduced resistance to lateral pushing or circling when the rat is placed on a flat surface, yet maintains normal posture at rest (2); spontaneous rotation to the left (3); and lack of spontaneous movement or unconsciousness (4).

### 3.4. Regional Cerebral Blood Flow

Laser Doppler flowmetry was used to monitor cerebral blood flow (CBF). This approach measures blood flow changes on a non-invasive intravenous level using a probe positioned on the skull above the MCA territory. Moving red blood cells generate a Doppler shift that can be detected by the laser Doppler flowmeter, which enables real-time, continuous measures of CBF.

### 3.5. Cytokine Assay

To determine the therapeutic effect of phellopterin on inflammation in rats challenged with cerebral I/R injury, we measured serum malondialdehyde (MDA), superoxide dismutase (SOD), and inflammatory cytokines [tumor necrosis factor-alpha (TNF-α) and IL-6]. After treatment, the blood sample was obtained from rats and placed at room temperature for 30 minutes for coagulation; the mixture was centrifuged at 3,000 rpm for 10 minutes to separate the serum. The sera were subsequently stored frozen at -80°C until analysis. Using ELISA kits, TNF-α (SRTA00, R&D Systems, USA), IL-6 (R6000B, R&D Systems, USA) cytokine levels, MDA levels (CEA597Ge, CLOUD-CLONE CORP., USA), and SOD activity (SES134Ra, CLOUD-CLONE CORP., USA) were measured by protocols provided by the manufacturer, and each sample was measured in triplicate for verification.

### 3.6. RNA Extraction and Real-time Polymerase Chain Reaction

**Figure 1.**
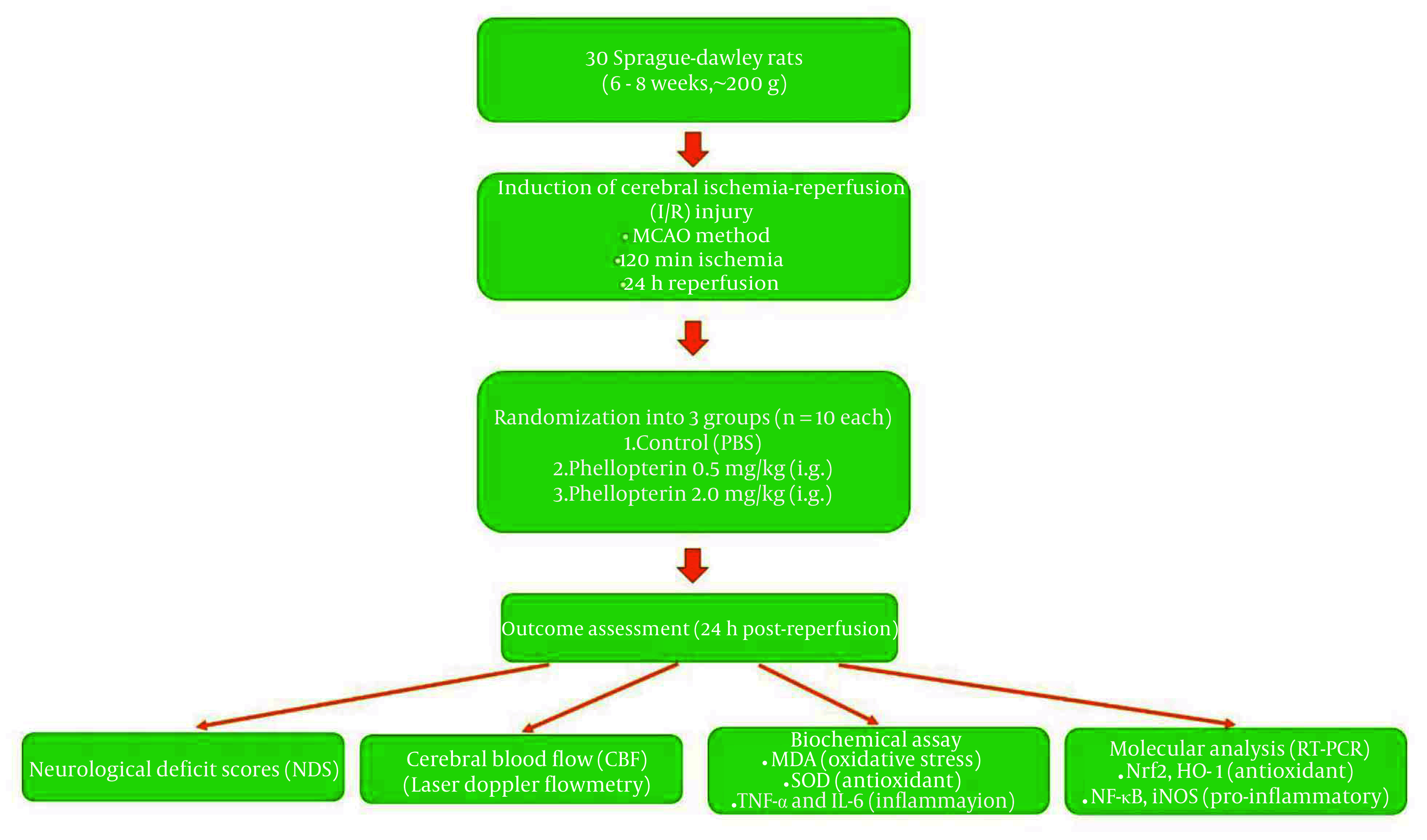
Flowchart of experimental design

Total RNA from the hippocampi of rats was extracted using a TRIzol reagent (15596018, Invitrogen, USA) according to the manufacturer’s protocol. RNA concentration and purity were determined by a NanoDrop spectrophotometer. One µg of total RNA was reverse-transcribed into complementary DNA (cDNA) using the High-Capacity cDNA Reverse Transcription Kit (4368814, Applied Biosystems, California, USA). Quantification of gene expression of nuclear factor erythroid 2-related factor 2 (Nrf2), heme oxygenase-1 (HO-1), NF-κB, and inducible nitric oxide synthase (iNOS) was performed using real-time PCR analysis (4364346, SYBR Green Master Mix, Applied Biosystems) and specific primers for each target gene. Reactions were carried out in 20 µL reaction systems including 10 µL SYBR Green master mix, 1 µL cDNA template, and 0.5 µM each primer. The PCR was conducted under cycling conditions of 10 min denaturation at 95°C, followed by 40 cycles of 15 s at 95°C and 1 min at 60°C for annealing/extension. The target genes’ relative expression levels were normalized to the housekeeping gene β-actin and processed with the ΔΔCt method. Assays were done in triplicate. The primer sequences used for the RT-qPCR assay are shown in Table 1. The experimental design of the study is presented in the flowchart in Figure 1. 

**Table 1.** The Primers Used for Real-time Polymerase Chain Reaction Assay

Genes and Primers	Sequences (5'→3')
**NF-κB**	
Forward	TGCAGAAAGAAGACATTGA
Reverse	AGGCTAGGGTCAGCGTATGG
**iNOS**	
Forward	TGTGTTCCACCAGGAGATGTTG
Reverse	GCTTCCGACTTTCCTGTCTC
**NEF2L2 (Nrf2)**	
Forward	TGATTTAAGCAGCATACAGCAG
Reverse	GTATTAAGACACTGTAACTCGGG
**HO-1**	
Forward	CAAGCGCTATGTTCAGCGAC
Reverse	GCTTGAACTTGGTGGCACTG
**β-actin**	
Forward	GCAGGAGTACGATGAGTCCG
Reverse	TGTCACCTTCACCGTTCCA G

Abbreviations: NF-κB, nuclear factor kappa B; iNOS, inducible nitric oxide synthase; Nrf2, nuclear factor erythroid 2-related factor 2; HO-1, heme oxygenase-1.

### 3.7. Statistical Analysis

Statistical analyses were performed using IBM SPSS Statistics software, version 26. Results were expressed as mean values with standard deviations, and statistical significance was determined at P-values below 0.05. Effect size measures were calculated to complement P-values: Cohen’s d for pairwise comparisons and eta squared (η^2^) for ANOVA analyses. Data visualization was accomplished using GraphPad Prism 8. For normally distributed datasets, one-way ANOVA followed by Tukey’s post hoc test was used. For datasets that did not meet normality assumptions (as assessed by the Shapiro-Wilk test), the Kruskal-Wallis test was applied, followed by Dunn’s test with Bonferroni correction for multiple comparisons. The NDS were analyzed using nonparametric tests due to their ordinal nature.

## 4. Results

### 4.1. Effects of Phellopterin on Neurological Deficit Scores in Cerebral Ischemia-Reperfusion Injury

**Figure 2.**
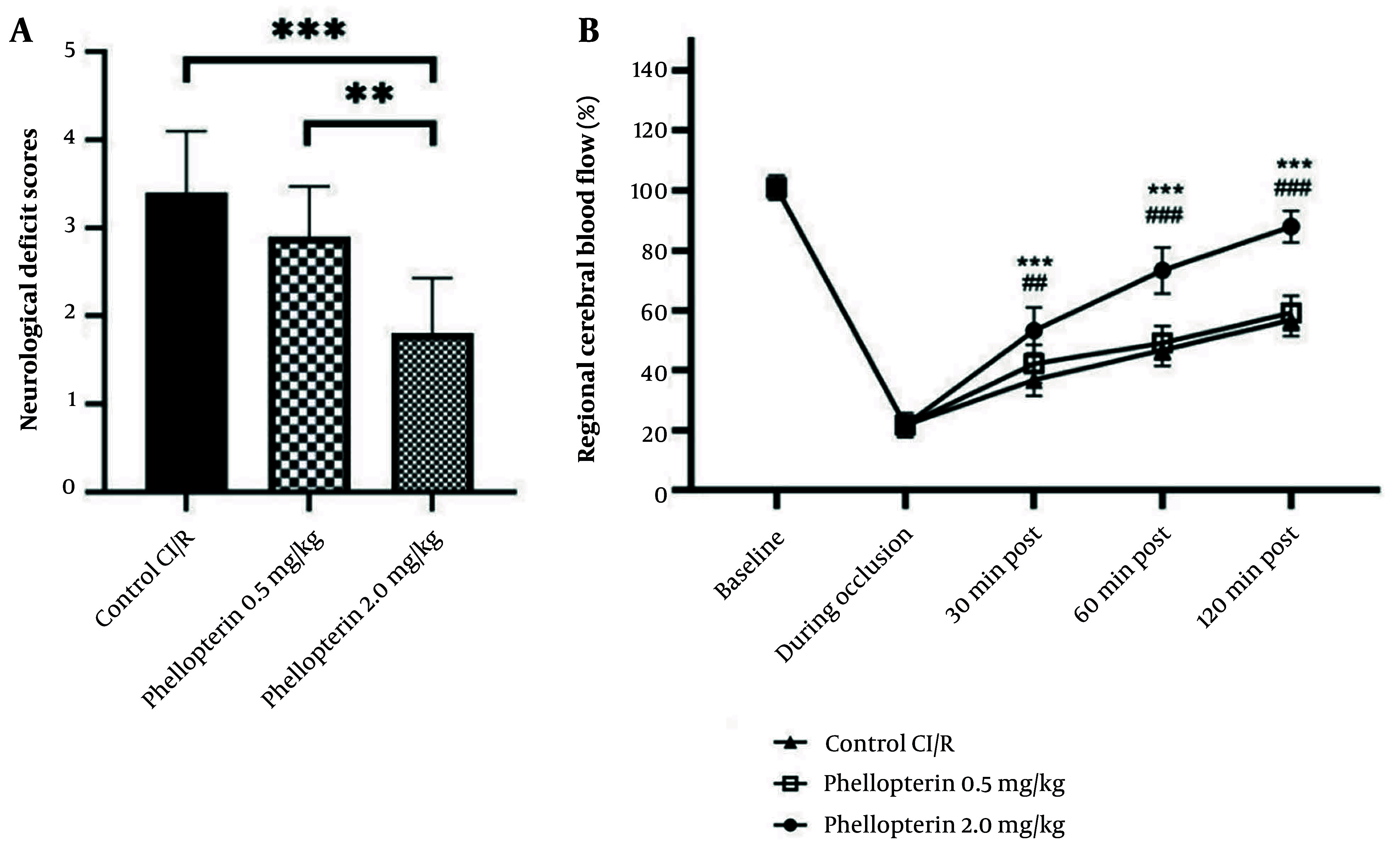
Effects of phellopterin on NDS and CBF following cerebral ischemia-reperfusion injury; A, the impact of phellopterin on NDS following cerebral I/R injury. Treatment with 2 mg/kg phellopterin significantly reduced NDS compared to both the control group and the 0.5 mg/kg phellopterin group; B, the effect of phellopterin on regional CBF in rats subjected to cerebral I/R injury. At a dose of 2.0 mg/kg, phellopterin led to a marked recovery of CBF at 30, 60, and 120 minutes post-reperfusion, nearing baseline levels. The data is presented as the mean ± standard deviation. Statistical significance is indicated as *** P < 0.001 and ** P < 0.01 vs control grouop, and ### P < 0.001 and ## P < 0.01 vs 0.5 mg/kg phellopterin group. (Abbreviations: NDS, neurological deficits score; CBF, cerebral blood flow; I/R, ischemia/reperfusion).

In our study, to evaluate the effects of phellopterin on NDS after cerebral I/R injury, we organized the following groups: The cerebral I/R control group received sterile PBS, the cerebral I/R group received 0.5 mg/kg phellopterin, and the cerebral I/R group received 2 mg/kg phellopterin, administered via the intragastric route. The NDS was assessed 24 hours after reperfusion. As seen in Figure 2A, 2 mg/kg phellopterin significantly reduced NDS compared to both control cerebral I/R (P < 0.0001) and 0.5 mg/kg phellopterin (P = 0.0017). Administration of higher doses of phellopterin can reverse NDS and present therapeutic effects against cerebral I/R injury.

### 4.2. Effects of Phellopterin on Regional Cerebral Blood Flow in Cerebral Ischemia-Reperfusion Injury

The data revealed that phellopterin promoted regional CBF impairment by cerebral I/R injury in rats. In the phellopterin group, a dose of 2.0 mg/kg significantly recovered CBF at 30 and 60 minutes after reperfusion, nearing baseline levels by 120 minutes post-reperfusion (P < 0.0001). Conversely, the vehicle group had sustained decreases in CBF during the duration of the reperfusion period. The 0.5 mg/kg phellopterin dose did trend toward increased CBF but was not as effective as the upper dose (Figure 2B). These findings suggest that phellopterin, particularly at 2.0 mg/kg, may have protective effects against cerebral I/R injury by enhancing blood flow recovery.

### 4.3. Effects of Phellopterin on Oxidative Stress Markers in Cerebral Ischemia-Reperfusion Injury

**Figure 3.**
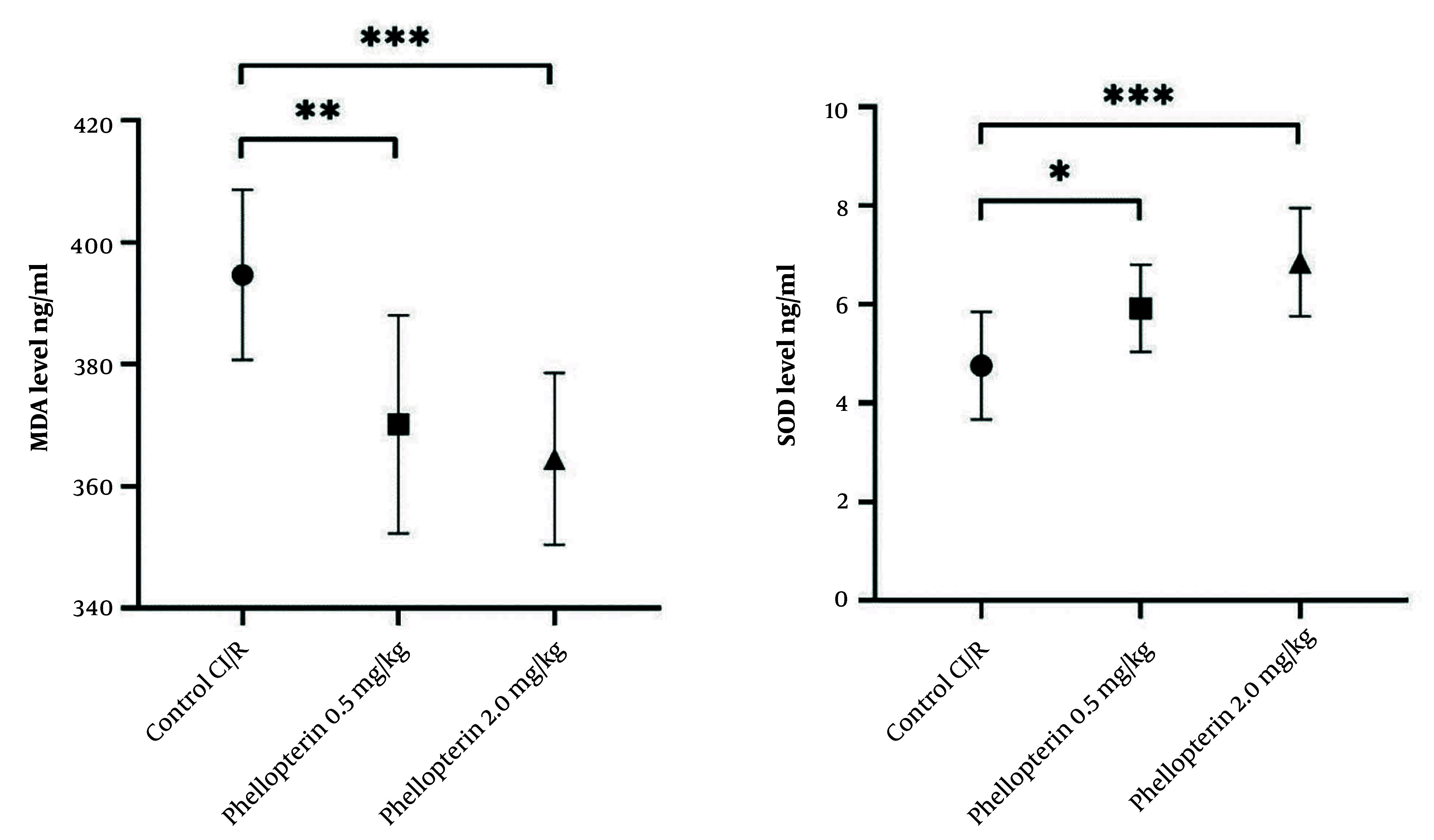
Antioxidant effects of phellopterin on oxidative stress markers following CI/R injury; The impact of phellopterin on markers of oxidative stress by determining the levels of MDA (ng/mL) and activity of SOD (ng/mL) in rats throughout CI/R injury. Compared to the control group, phellopterin-treated groups (0.5 mg/kg and 2 mg/kg) showed marked reductions in MDA levels and significantly higher SOD activity. Each group consisted of n = 10 rats. The data is presented as the mean ± standard deviation. Statistical analysis was performed using one-way ANOVA followed by Tukey’s post hoc test. Statistical significance is indicated as *** P < 0.001, ** P < 0.01, and * P < 0.05 (Abbreviations: CI/R, cerebral ischemia/reperfusion; MDA, malondialdehyde; SOD, superoxide dismutase).

The role of phellopterin against oxidative stress markers after cerebral I/R injury was investigated. Treatment with 0.5 mg/kg of phellopterin reduced MDA levels, a marker of lipid peroxidation, compared to the control cerebral I/R injury group (P = 0.0039). On the other hand, the lowest level of MDA was found in the group treated with 2 mg/kg phellopterin (P = 0.0005), denoting stronger antioxidant capabilities at this dosage. Similarly, SOD activity was markedly elevated in the group treated with 0.5 mg/kg phellopterin compared with control (P = 0.04). Treatment with 2 mg/kg phellopterin led to a significant increase in SOD activity (P = 0.0003), suggesting that this phellopterin dose provided a stronger enhancement of antioxidant defense mechanisms (Figure 3). 

### 4.4. Effects of Phellopterin on Nuclear Factor Erythroid 2-Related Factor 2 and Heme Oxygenase-1 Expression in Cerebral Ischemia-Reperfusion Injury

**Figure 4.**
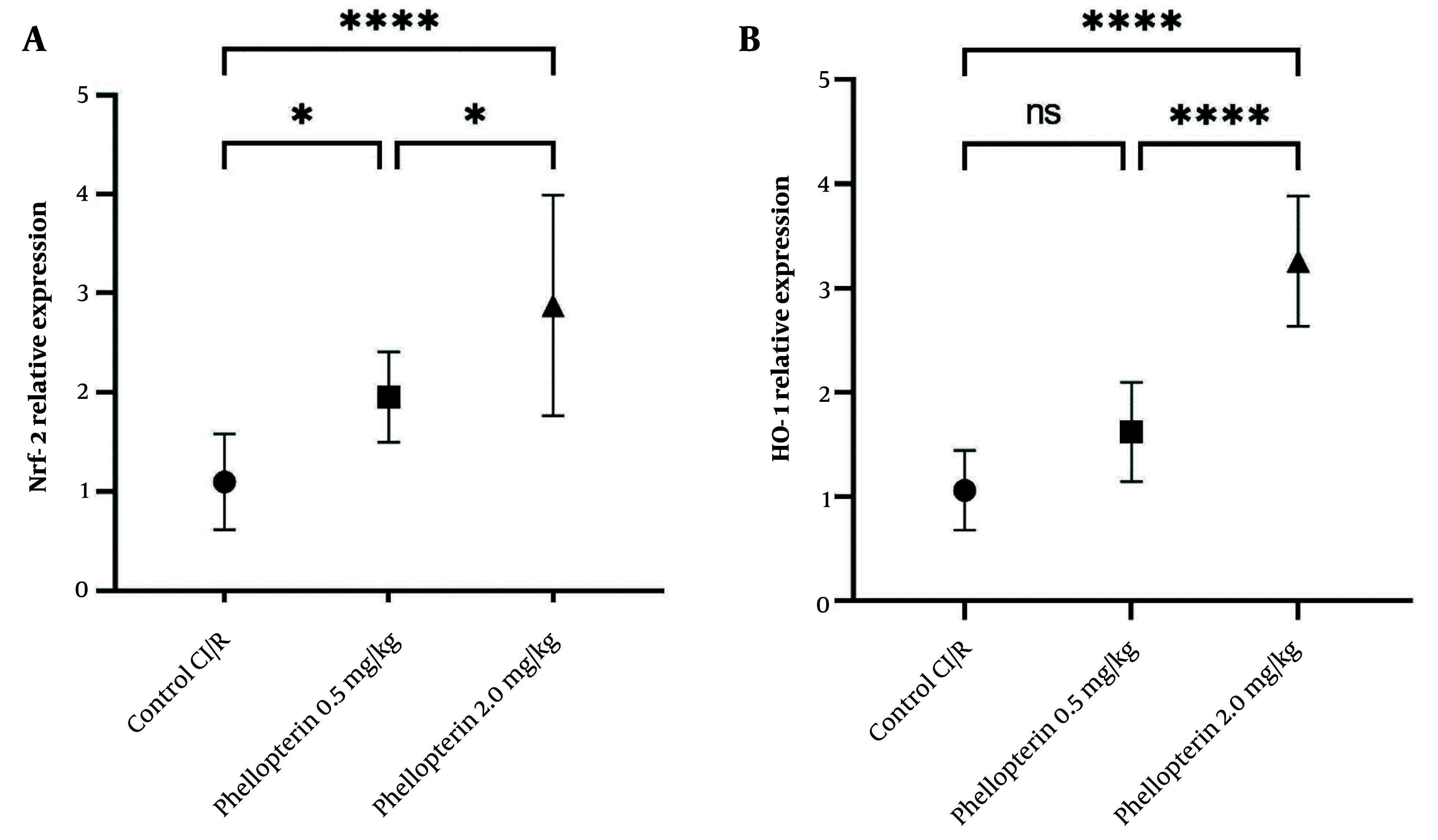
Effects of phellopterin on A, Nrf2 and B, HO-1 expression in cerebral I/R injury; The expression levels of Nrf2 and HO-1 were increased after phellopterin treatment, especially at the dose of 2 mg/kg, compared with the control group. Each group included n = 10 rats. Data are expressed as mean ± standard deviation. Statistical significance is indicated as **** P < 0.0001 and * P < 0.05 (Abbreviations: CI/R, cerebral ischemia/reperfusion; Nrf2, nuclear factor erythroid 2-related factor 2; HO-1, heme oxygenase-1).

The expression levels of Nrf2 and HO-1 were also assessed to understand the protective mechanisms of phellopterin against cerebral I/R injury. Following treatment with 0.5 mg/kg phellopterin, Nrf2 levels were mildly elevated (P = 0.04), demonstrating a low-level protective effect at this dose. However, in the experimental group receiving 2 mg/kg of phellopterin, Nrf2 expression was significantly increased (P < 0.0001), indicating a strong activation of cellular defense mechanisms against oxidative stress and inflammation. In addition, phellopterin at 2 mg/kg significantly increased Nrf2 expression compared to the 0.5 mg/kg group. The administration of 0.5 mg/kg of phellopterin increased HO-1 expression (P = 0.05), which was not statistically significant compared to the control group. In contrast, treatment with 2 mg/kg of phellopterin led to a significant elevation in HO-1 expression (P < 0.0001) (Figure 4). These results collectively indicate that a higher concentration of phellopterin can activate neuroprotective pathways against cerebral I/R injury.

### 4.5. Impact of Phellopterin on Inflammatory Cytokines Following Cerebral Ischemia-Reperfusion Injury

**Figure 5.**
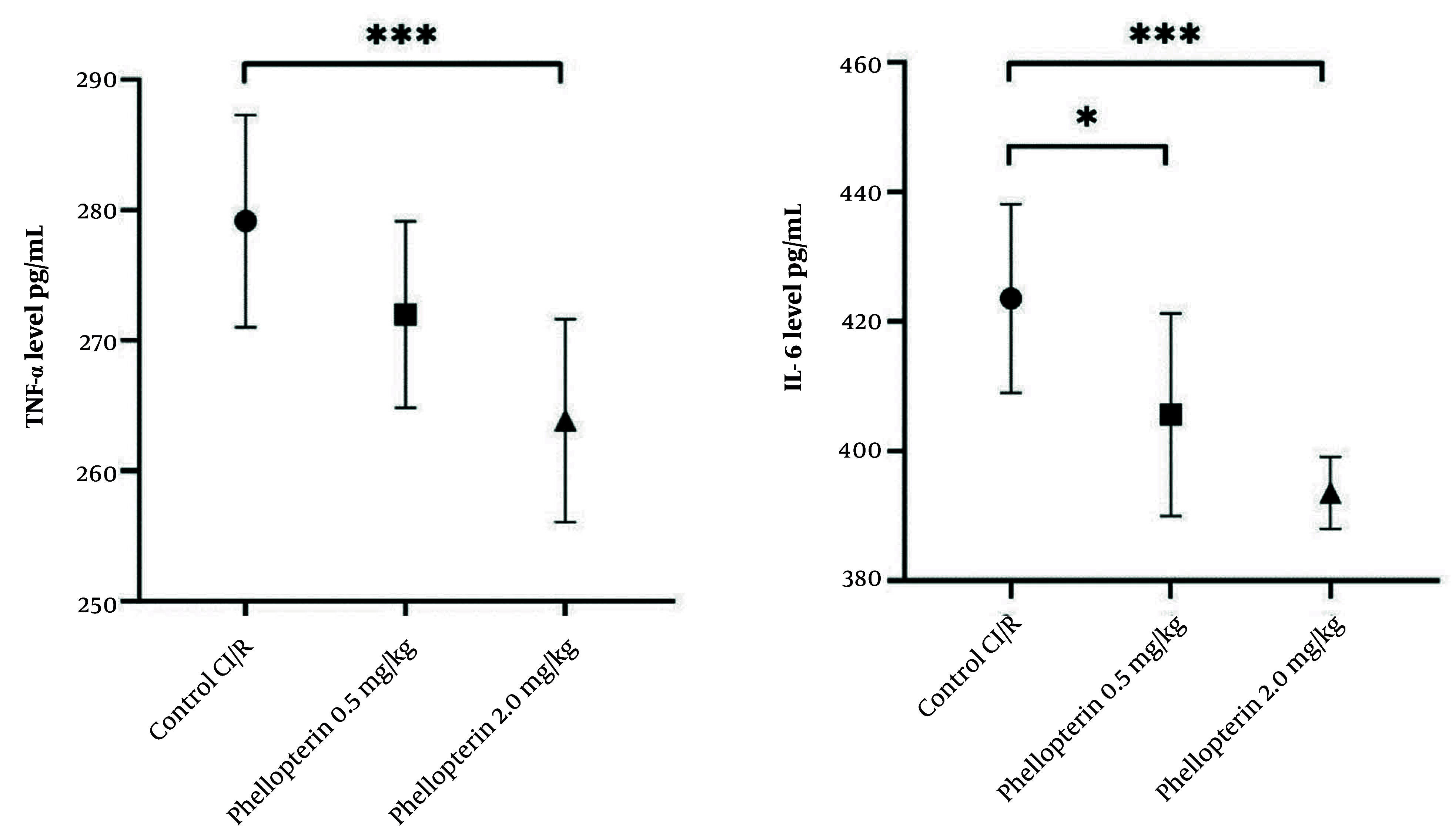
Anti-inflammatory effects of phellopterin on cerebral I/R injury; The effects of phellopterin on pro-inflammatory cytokines, TNF-α (pg/mL), and IL-6 (pg/mL) in rats after CI/R injury. Phellopterin treatment at both 0.5 mg/kg as well as 2 mg/kg significantly reduced the release of TNF-α and IL-6, with the 2 mg/kg dose providing greater reduction in inflammatory cytokines. Each group included n = 10 rats. Data are expressed as mean ± standard deviation. Statistical analysis was conducted using one-way ANOVA followed by Tukey’s multiple comparisons test. Statistical significance is indicated as *** P < 0.001 and * P < 0.05 (Abbreviations: CI/R, cerebral ischemia/reperfusion; TNF-α, tumor necrosis factor-alpha; IL-6, interleukin-6).

The effects of phellopterin on inflammatory cytokines were also examined. Treatment with 0.5 mg/kg of phellopterin resulted in a moderate decrease in TNF-α levels, although this change was not statistically significant (P = 0.11). Comparatively, the 2 mg/kg of phellopterin dose decreased TNF-α levels significantly (P = 0.0004), suggesting a more potent anti-inflammatory response at a higher dosage. Furthermore, treatment with 0.5 mg/kg of phellopterin led to a significant reduction in IL-6 levels (P = 0.01) when compared to the control group. In the 2 mg/kg phellopterin treatment group, IL-6 was markedly reduced (P < 0.0001), demonstrating its potent anti-inflammatory potential (Figure 5). 

### 4.6. Effects of Phellopterin on Nuclear Factor Kappa B and Inducible Nitric Oxide Synthase Expression in Cerebral Ischemia-Reperfusion Injury

**Figure 6.**
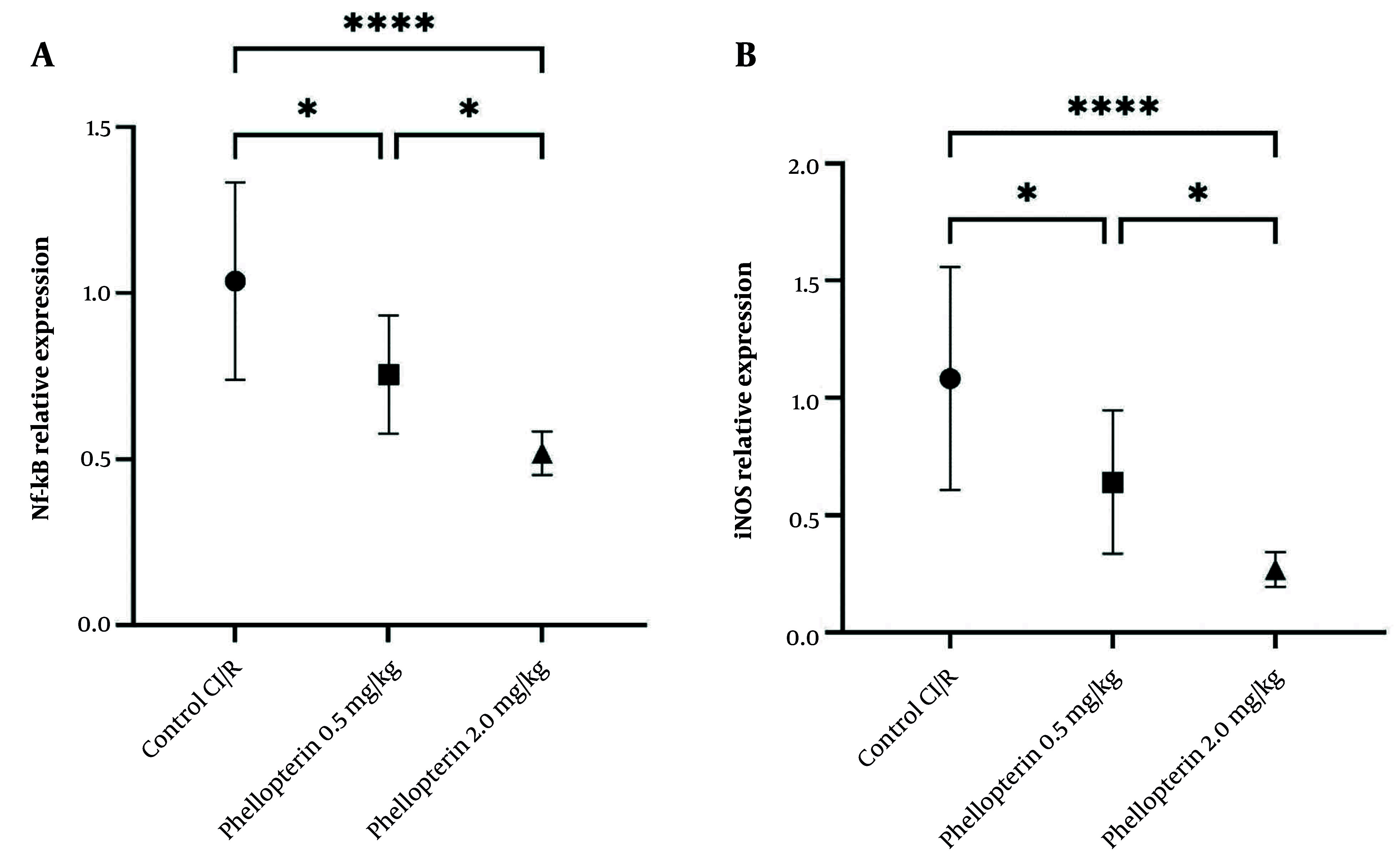
Effects of phellopterin on A, NF-κB and B, iNOS expression in cerebral I/R injury; The expression levels of Nf-κB and iNOS were decreased after phellopterin treatment, especially at the dose of 2 mg/kg, compared with the control group. Each group included n = 10 rats. Data are expressed as mean ± standard deviation. Statistical significance is indicated as **** P < 0.0001 and * P < 0.05 (Abbreviations: CI/R, cerebral ischemia/reperfusion; NF-κB, nuclear factor-kappa B; iNOS, inducible nitric oxide synthase).

The expression levels of NF-κB and iNOS were assessed to gain further insight into the anti-inflammatory effects of phellopterin after cerebral I/R injury. Treatment with 0.5 mg/kg of phellopterin led to a moderate decrease in NF-κB expression (P = 0.01). By contrast, NF-κB expression was significantly inhibited in the group treated with 2 mg/kg phellopterin (control, P < 0.0001). This indicates that the high doses are potent inhibitors of inflammatory signaling pathways. Similarly, treatment with 0.5 mg/kg of phellopterin resulted in a reduction in iNOS levels (P = 0.01). With administration of 2 mg/kg phellopterin, iNOS levels decreased further (P < 0.0001). The results showed that the treatment with 2 mg/kg of phellopterin remarkably decreased the expression of iNOS more than did the 0.5 mg/kg of phellopterin, proving the stronger anti-inflammatory effect of the higher dose (Figure 6). These results collectively indicate that phellopterin exerts significant beneficial effects on both oxidative stress and inflammatory markers following cerebral I/R injury, with the 2 mg/kg dosage demonstrating superior efficacy compared to lower doses and control treatments.

## 5. Discussion

The current study elucidated the neuroprotective role of phellopterin in cerebral I/R injury in rats. Due to its role in reducing oxidative stress and regulating inflammatory mechanisms, phellopterin may serve as a promising candidate for ischemic stroke therapy, a clinical condition marked by intricate pathophysiological cascades encompassing oxidative injury, neuroinflammation, and neuronal apoptosis. Previous work on phellopterin has focused on peripheral inflammatory disorders and neurodegenerative models, but its impact on acute cerebrovascular injury has not been explored. Our findings extend phellopterin’s therapeutic profile from chronic and peripheral pathologies to acute central nervous system injury, demonstrating its ability to modulate both oxidative and inflammatory pathways in the context of cerebral I/R injury.

The pathogenesis of cerebral I/R injury is primarily driven by the overproduction of reactive oxygen species (ROS), which generates oxidative stress and induces the damage of lipids, proteins, and nucleic acids in neural tissue (17, 18). In this study, phellopterin significantly reduced MDA, a marker of lipid peroxidation, and increased the activity of SOD, a vital antioxidant enzyme. This dual effect on oxidative stress markers reflects phellopterin’s ability to counteract the deleterious impact of ROS, a hallmark of I/R injury. The upregulation of Nrf2 and its downstream target HO-1 seen in this study also illuminates phellopterin’s antioxidant capacity. The Nrf2 is a master regulator of cellular antioxidant response, and its activation has been shown to enhance cellular defenses against oxidative damage (19). Since the expression of Nrf2 and HO-1 helps to mitigate ROS-mediated apoptosis of neurons, which is a mechanism that drives the I/R injury, phellopterin likely protects neurons by promoting the expression of Nrf2 and HO-1.

These findings are consistent with previous research on natural compounds known for their antioxidant properties. Other studies involving phytochemicals, such as resveratrol and curcumin, have also demonstrated the ability to upregulate Nrf2 and enhance antioxidant defenses within cells (20, 21). Although the precise mechanism by which phellopterin activates Nrf2 remains undefined, evidence from the coumarin literature offers plausible hypotheses. Many naturally occurring coumarins possess electrophilic centers capable of modifying cysteine residues on Kelch-like ECH-associated protein 1 (Keap1), leading to the dissociation of Nrf2 from the Keap1-Nrf2 complex and subsequent nuclear translocation. This allows Nrf2 to bind antioxidant response elements (AREs) and initiate transcription of cytoprotective genes, such as HO-1 and NQO1 (22, 23).

Additionally, several coumarins, such as esculetin and imperatorin, have been shown to activate upstream signaling pathways including phosphoinositide 3-kinase/protein kinase B (PI3K/Akt) and mitogen-activated protein kinases (MAPKs), particularly ERK1/2 and JNK. These kinase pathways can phosphorylate Nrf2, enhancing its nuclear accumulation independent of Keap1 oxidation (24, 25). Whether phellopterin acts through Keap1-cysteine modification, kinase-mediated phosphorylation, or a combination of both remains to be elucidated in future mechanistic studies. Although coumarins share structural motifs that enable Nrf2 activation, variations such as methoxy and furan substitutions in phellopterin may alter lipophilicity, metabolic stability, and protein-binding properties. These differences could impact its bioactivity and distinguish its mechanism from related coumarins such as esculetin or imperatorin. Therefore, while findings from other coumarins provide useful context, direct extrapolation to phellopterin should be made with caution.

Another important contributor to cerebral I/R injury is neuroinflammation, which further damages neurons via the secretion of pro-inflammatory cytokines and microglial activation (26). This study demonstrated that phellopterin significantly reduced levels of TNF-α and IL-6, the two major pro-inflammatory cytokines involved in the pathogenesis of ischemic stroke. The inhibition of the nuclear factor, NF-κB, a key transcription factor in the inflammatory response, reinforces phellopterin’s anti-inflammatory potential.

This anti-inflammatory profile is extended by the observed reduction of iNOS expression. Increased expression of iNOS after I/R injury leads to excessive endogenously produced nitric oxide (NO), which in turn reacts with superoxide to generate peroxynitrite, an extremely active and injurious molecule (27). Phellopterin most probably inhibits the generation of these toxic species through iNOS inhibition in neurons, consequently protecting the neuronal cells. These findings are mostly consistent with previous studies of phellopterin in other disease contexts (such as CAC and diabetic ulcers), showing phellopterin-mediated attenuation of inflammation (10, 12). For example, Xu et al. reported TLR4/NF-κB suppression by phellopterin in models of colitis, a mechanism that could be relevant to its effects on I/R injury in the brain as well (12). This phenomenon supports our hypothesis that phellopterin is a well-rounded anti-inflammatory agent for diseases manifesting with diverse features of inflammation or ischemia.

The superior efficacy of the 2 mg/kg dose of phellopterin compared to the 0.5 mg/kg dose highlights the importance of dose optimization in therapeutic interventions. Higher doses of phellopterin resulted in greater reductions in oxidative stress markers, more pronounced suppression of pro-inflammatory cytokines, and stronger upregulation of Nrf2 and HO-1. This dose-dependent effect is in line with pharmacological principles, where higher concentrations of active compounds often achieve more substantial therapeutic effects, provided that toxicity thresholds are not exceeded. Importantly, the safety profile of phellopterin inferred from its prior use in traditional medicine suggests feasibility for higher-dose scheduling (8, 28). Nevertheless, chronic administration of this compound will be required to further characterize its toxicity and pharmacokinetics. This is particularly important for bridging from preclinical discoveries to clinical applications, which requires scrutiny between efficacy and safety.

Stroke is one of the leading causes of long-term disability and mortality worldwide, and it has few therapeutic options. Novel approaches to preventing or attenuating I/R injury itself may enhance the effectiveness of existing therapies, such as thrombolytics and mechanical thrombectomy, both of which aim to restore blood flow to ischemic tissues but leave the secondary damage by I/R injury unmet (29). These results indicate that phellopterin may serve as an additional therapeutic targeting oxidative or inflammatory cascades.

Moreover, the natural origin of phellopterin and its established safety profile in preclinical models make it a particularly attractive candidate for further development. Its ability to target multiple pathways simultaneously — oxidative stress, inflammation, and apoptosis — gives it an edge over single-target drugs, which may fail to address the multifaceted nature of I/R injury. Compared to other neuroprotective agents evaluated in cerebral I/R injury, such as edaravone, curcumin, and minocycline, phellopterin appears to exhibit a similarly multi-targeted mode of action. Edaravone, a free radical scavenger approved for clinical use in Japan for acute ischemic stroke, primarily exerts antioxidant effects by neutralizing hydroxyl and peroxyl radicals, but its anti-inflammatory efficacy is relatively limited (30, 31). Curcumin, a natural polyphenol, has been shown to activate Nrf2 signaling while concurrently inhibiting pro-inflammatory mediators such as NF-κB, making it an effective agent against oxidative and inflammatory damage (32, 33). Minocycline, a tetracycline antibiotic, is widely recognized for its neuroprotective and anti-inflammatory properties, especially through microglial inhibition and suppression of cytokines like TNF-α and IL-1β, but its direct antioxidant effects are less prominent (34, 35).

Compared with these agents, phellopterin (a coumarin derivative) demonstrated both upregulation of Nrf2/HO-1 and suppression of NF-κB/iNOS in our acute I/R model, consistent with a dual antioxidant-anti-inflammatory profile. This pattern is mechanistically closest to curcumin (activation of Nrf2 and inhibition of NF-κB), but phellopterin likely engages different molecular triggers: Coumarins have been reported to modulate the Keap1/Nrf2 axis via direct (electrophilic) modification of Keap1 cysteines or indirectly via upstream kinase pathways (e.g., PI3K/Akt, MAPKs) (36). By contrast, edaravone acts predominantly via radical scavenging, while minocycline appears to act primarily through modulation of innate immune cells and anti-apoptotic signaling rather than direct activation of Nrf2.

Importantly, the present work is an acute, 24-hour study and does not establish comparative pharmacodynamics or safety profiles; head-to-head experiments and pharmacokinetic/pharmacodynamic studies will be required to position phellopterin relative to these established compounds for stroke therapy (37, 38). The therapeutic landscape for ischemic stroke currently centers on reperfusion strategies such as thrombolysis and mechanical thrombectomy. However, these interventions do not directly address the secondary oxidative and inflammatory cascades that exacerbate neuronal damage (39, 40). Agents with dual antioxidant and anti-inflammatory properties, such as phellopterin, could serve as adjunctive therapies to limit reperfusion injury and improve overall outcomes. By targeting both oxidative stress and inflammation, phellopterin aligns with a multi-targeted therapeutic approach that may complement and enhance the benefits of existing treatments (39).

While the present findings are promising, they should be interpreted with caution. Further validation in large-animal stroke models and chronic studies assessing long-term neurological outcomes is essential before clinical translation can be considered (41, 42). Such studies will be critical for assessing reproducibility, safety, and dose optimization in more clinically relevant settings. Future investigations should include pharmacokinetic profiling to define bioavailability and brain penetration, chronic dosing safety assessments, combination studies with standard-of-care therapies (e.g., tissue plasminogen activator), and deeper mechanistic analyses focusing on key signaling pathways, including Keap1/Nrf2, PI3K/Akt, and mitogen-activated protein kinase (MAPK) cascades (43, 44). These steps will be crucial to fully establish phellopterin’s therapeutic potential and guide its path toward translational application.

Several limitations of this study should be acknowledged. First, outcomes were assessed only at 24 h post-reperfusion, representing the acute phase of cerebral I/R injury. This design allowed us to capture early molecular and functional changes, but does not provide information on the durability of phellopterin’s effects. Long-term studies evaluating survival, infarct evolution, and functional recovery over 7 - 28 days — including motor and cognitive behavioral tests such as the rotarod and Morris water maze — are warranted to determine sustained neuroprotection. Second, we did not perform histopathological analyses such as infarct volume measurement by 2,3,5-triphenyltetrazolium chloride (TTC) staining or neuronal apoptosis assessment by terminal deoxynucleotidyl transferase dUTP nick end labeling (TUNEL). Correlating molecular findings with structural brain injury will be an important next step to strengthen the translational relevance of our data. Third, while the rat MCAO model is well established and reproducible, it does not fully replicate the complexity of human ischemic stroke, particularly with respect to patient heterogeneity, comorbidities, vascular anatomy, and immune responses. Additionally, interspecies differences in drug metabolism may affect efficacy and safety profiles. Large-animal models and eventual randomized clinical trials will be required to determine whether the benefits observed here can be translated to human stroke therapy.

Mechanistic exploration of the Keap1/Nrf2 axis, along with signaling intermediates like PI3K/Akt and MAPK pathways, using western blotting or immunofluorescence, would offer greater clarity into how phellopterin modulates cellular stress responses. These directions will help further define the therapeutic potential and molecular specificity of phellopterin in ischemic stroke.

To our knowledge, this is the first study to demonstrate the neuroprotective potential of phellopterin in an in vivo model of cerebral I/R injury. The dual modulation of antioxidant (Nrf2/HO-1) and inflammatory (NF-κB/iNOS) pathways highlights its capacity to target multiple pathological mechanisms simultaneously, which is a desirable feature in stroke therapeutics. However, to strengthen its translational relevance, future studies should include comparative evaluations against standard agents such as edaravone, curcumin, or minocycline, which have well-characterized efficacy in similar models. Additionally, exploring combination strategies — for example, phellopterin co-administered with thrombolytics or neuroregenerative compounds — may help determine its synergistic potential and further elevate its therapeutic significance. Future studies could also investigate phellopterin in combination with thrombolytic agents (e.g., tPA) or established neuroprotectants to assess potential synergistic benefits. Another important area requiring investigation is the pharmacokinetic profile of phellopterin. Data on absorption, bioavailability, brain penetration, and metabolism are currently lacking. These studies will be critical to determine whether effective concentrations can be achieved in the brain and to assess translational feasibility.

### 5.1. Conclusions

This study provides the first evidence to support the neuroprotective effects of phellopterin in experimental cerebral I/R injury. Phellopterin promotes neurological recovery and decreases neuron injury by reducing oxidative stress and inflammation. However, additional research is required to validate these findings in chronic and translational contexts. Future studies should include extended survival assessments and exploration in higher-order preclinical models to better assess its therapeutic viability in humans.

## Data Availability

The dataset presented in the study is available on request from the corresponding author during submission or after publication. The data are not publicly available due to privacy and institutional limitations.

## References

[1] G. B. D. Causes of Death Collaborators (2024). Global burden of 288 causes of death and life expectancy decomposition in 204 countries and territories and 811 subnational locations, 1990-2021: a systematic analysis for the Global Burden of Disease Study 2021.. Lancet..

[2] Feigin VL, Brainin M, Norrving B, Martins SO, Pandian J, Lindsay P (2025). World Stroke Organization: Global Stroke Fact Sheet 2025.. Int J Stroke..

[3] Li C, Sun G, Chen B, Xu L, Ye Y, He J (2021). Nuclear receptor coactivator 4-mediated ferritinophagy contributes to cerebral ischemia-induced ferroptosis in ischemic stroke.. Pharmacol Res..

[4] Li M, Han B, Zhao H, Xu C, Xu D, Sieniawska E (2022). Biological active ingredients of Astragali Radix and its mechanisms in treating cardiovascular and cerebrovascular diseases.. Phytomedicine..

[5] Sun Y, Yang X, Xu L, Jia M, Zhang L, Li P (2023). The Role of Nrf2 in Relieving Cerebral Ischemia-Reperfusion Injury.. Curr Neuropharmacol..

[6] Li W, Tan C, Liu Y, Liu X, Wang X, Gui Y (2015). Resveratrol ameliorates oxidative stress and inhibits aquaporin 4 expression following rat cerebral ischemia-reperfusion injury.. Mol Med Rep..

[7] Yang C, Zhang X, Fan H, Liu Y (2009). Curcumin upregulates transcription factor Nrf2, HO-1 expression and protects rat brains against focal ischemia.. Brain Res..

[8] Han HS, Jeon H, Kang SC (2018). Phellopterin isolated from Angelica dahurica reduces blood glucose level in diabetic mice.. Heliyon..

[9] Liang WH, Chang TW, Charng YC (2018). Effects of drying methods on contents of bioactive compounds and antioxidant activities of Angelica dahurica.. Food Sci Biotechnol..

[10] Zou J, Duan Y, Wang Y, Liu A, Chen Y, Guo D (2022). Phellopterin cream exerts an anti-inflammatory effect that facilitates diabetes-associated cutaneous wound healing via SIRT1.. Phytomedicine..

[11] Bartnik M (2024). Methoxyfuranocoumarins of Natural Origin-Updating Biological Activity Research and Searching for New Directions-A Review.. Curr Issues Mol Biol..

[12] Xu X, Su Y, Pan Y, Shen M, Liu D, Liu Z (2023). The therapeutic effect of phellopterin on colitis-associated cancer and its effects on TLR4/NF-kappaB pathway and macrophage M2 polarization.. Cell Mol Biol (Noisy-le-grand)..

[13] Chen X, Zhang Y, Pei J, Zeng X, Yang Y, Zhang Y (2022). Phellopterin alleviates atopic dermatitis-like inflammation and suppresses IL-4-induced STAT3 activation in keratinocytes.. Int Immunopharmacol..

[14] Takomthong P, Waiwut P, Yenjai C, Sripanidkulchai B, Reubroycharoen P, Lai R (2020). Structure-Activity Analysis and Molecular Docking Studies of Coumarins from Toddalia asiatica as Multifunctional Agents for Alzheimer's Disease.. Biomedicines..

[15] Gong L, Tang Y, An R, Lin M, Chen L, Du J (2017). RTN1-C mediates cerebral ischemia/reperfusion injury via ER stress and mitochondria-associated apoptosis pathways.. Cell Death Dis..

[16] Bederson JB, Pitts LH, Tsuji M, Nishimura MC, Davis RL, Bartkowski H (1986). Rat middle cerebral artery occlusion: evaluation of the model and development of a neurologic examination.. Stroke..

[17] Kong J, Chu R, Wen J, Yu H, Liu J, Sun Y (2024). Reactive oxygen species-responsive nanotherapy for the prevention and treatment of cerebral ischemia–reperfusion injury.. Chem Engin J..

[18] Olmez I, Ozyurt H (2012). Reactive oxygen species and ischemic cerebrovascular disease.. Neurochem Int..

[19] Vomund S, Schafer A, Parnham MJ, Brune B, von Knethen A (2017). Nrf2, the Master Regulator of Anti-Oxidative Responses.. Int J Mol Sci..

[20] Ghareghomi S, Rahban M, Moosavi-Movahedi Z, Habibi-Rezaei M, Saso L, Moosavi-Movahedi AA (2021). The Potential Role of Curcumin in Modulating the Master Antioxidant Pathway in Diabetic Hypoxia-Induced Complications.. Molecules..

[21] Wang X, Yuan Q, Xiao Y, Cai X, Yang Z, Zeng W (2024). Pterostilbene, a Resveratrol Derivative, Improves Ovary Function by Upregulating Antioxidant Defenses in the Aging Chickens via Increased SIRT1/Nrf2 Expression.. Antioxidants (Basel)..

[22] Taguchi K, Motohashi H, Yamamoto M (2011). Molecular mechanisms of the Keap1-Nrf2 pathway in stress response and cancer evolution.. Genes Cells..

[23] Krajka-Kuzniak V, Paluszczak J, Baer-Dubowska W (2017). The Nrf2-ARE signaling pathway: An update on its regulation and possible role in cancer prevention and treatment.. Pharmacol Rep..

[24] Zhou Y, Zheng J, Li Y, Xu DP, Li S, Chen YM (2016). Natural Polyphenols for Prevention and Treatment of Cancer.. Nutrients..

[25] Dinkova-Kostova AT, Kostov RV, Kazantsev AG (2018). The role of Nrf2 signaling in counteracting neurodegenerative diseases.. FEBS J..

[26] Jurcau A, Simion A (2021). Neuroinflammation in Cerebral Ischemia and Ischemia/Reperfusion Injuries: From Pathophysiology to Therapeutic Strategies.. Int J Mol Sci..

[27] Liu H, Li J, Zhao F, Wang H, Qu Y, Mu D (2015). Nitric oxide synthase in hypoxic or ischemic brain injury.. Rev Neurosci..

[28] Guo A, Lin J, Zhong P, Chen J, Wang L, Lin X (2023). Phellopterin attenuates ovarian cancer proliferation and chemoresistance by inhibiting the PU.1/CLEC5A/PI3K-AKT feedback loop.. Toxicol Appl Pharmacol..

[29] Stoll G, Nieswandt B, Schuhmann MK (2024). Ischemia/reperfusion injury in acute human and experimental stroke: focus on thrombo-inflammatory mechanisms and treatments.. Neurol Res Pract..

[30] Kikuchi K, Tancharoen S, Takeshige N, Yoshitomi M, Morioka M, Murai Y (2013). The efficacy of edaravone (radicut), a free radical scavenger, for cardiovascular disease.. Int J Mol Sci..

[31] Yamashita T, Abe K (2024). Update on Antioxidant Therapy with Edaravone: Expanding Applications in Neurodegenerative Diseases.. Int J Mol Sci..

[32] Zhang L, Han Y, Wu X, Chen B, Liu S, Huang J (2023). Research progress on the mechanism of curcumin in cerebral ischemia/reperfusion injury: a narrative review.. Apoptosis..

[33] Fan F, Lei M (2022). Mechanisms Underlying Curcumin-Induced Neuroprotection in Cerebral Ischemia.. Front Pharmacol..

[34] Plane JM, Shen Y, Pleasure DE, Deng W (2010). Prospects for minocycline neuroprotection.. Arch Neurol..

[35] Garrido-Mesa N, Zarzuelo A, Galvez J (2013). Minocycline: far beyond an antibiotic.. Br J Pharmacol..

[36] Hassanein EHM, Sayed AM, Hussein OE, Mahmoud AM (2020). Coumarins as Modulators of the Keap1/Nrf2/ARE Signaling Pathway.. Oxid Med Cell Longev..

[37] Cha SJ, Kim K (2022). Effects of the Edaravone, a Drug Approved for the Treatment of Amyotrophic Lateral Sclerosis, on Mitochondrial Function and Neuroprotection.. Antioxidants (Basel)..

[38] Zhang R, Yong VW, Xue M (2022). Revisiting Minocycline in Intracerebral Hemorrhage: Mechanisms and Clinical Translation.. Front Immunol..

[39] Chamorro A, Dirnagl U, Urra X, Planas AM (2016). Neuroprotection in acute stroke: targeting excitotoxicity, oxidative and nitrosative stress, and inflammation.. Lancet Neurol..

[40] Durukan A, Tatlisumak T (2007). Acute ischemic stroke: overview of major experimental rodent models, pathophysiology, and therapy of focal cerebral ischemia.. Pharmacol Biochem Behav..

[41] Howells DW, Sena ES, Macleod MR (2014). Bringing rigour to translational medicine.. Nat Rev Neurol..

[42] Saver JL (2006). Time is brain--quantified.. Stroke..

[43] O'Collins VE, Macleod MR, Donnan GA, Horky LL, van der Worp BH, Howells DW (2006). 1,026 experimental treatments in acute stroke.. Ann Neurol..

[44] Dinkova-Kostova AT, Kostov RV (2012). Glucosinolates and isothiocyanates in health and disease.. Trends Mol Med..

